# Evaluation of Decision Fusion Methods for Multimodal Biometrics in the Banking Application

**DOI:** 10.3390/s22062356

**Published:** 2022-03-18

**Authors:** Piotr Szczuko, Arkadiusz Harasimiuk, Andrzej Czyżewski

**Affiliations:** Faculty of Electronics Telecommunications and Informatics, Gdansk University of Technology, 80-233 Gdańsk, Poland; arkadiusz.harasimiuk@pg.edu.pl (A.H.); andczyze@pg.edu.pl (A.C.)

**Keywords:** biometry, multimodal biometrics, identity verification, data fusion, biometric systems, biometric sensors

## Abstract

An evaluation of decision fusion methods based on Dempster-Shafer Theory (DST) and its modifications is presented in the article, studied over real biometric data from the engineered multimodal banking client verification system. First, the approaches for multimodal biometric data fusion for verification are explained. Then the proposed implementation of comparison scores fusion is presented, including details on the application of DST, required modifications, base probability, and mass conversions. Next, the biometric verification process is described, and the engineered biometric banking system principles are provided. Finally, the validation results of three fusion approaches on synthetic and real data are presented and discussed, considering the desired outcome manifested by minimized false non-match rates for various assumed thresholds and biometric verification techniques.

## 1. Introduction

Biometric verification can be performed on the basis of many techniques. Systems and applications exist, exploiting one or many of the following: contact methods for fingerprint, hand vein, signature, and contactless: retina scan, voice, face image, 3D face geometry, and other traits. The convenience of use, speed, and reliability [1,2,3] are crucial in identity verification applications, as much as the critical condition: the user’s ability to handle the selected modality. Our work focuses on developing and implementing a multimodal biometric verification system, tested in real banking conditions, able to adjust to the changes of availability of the modalities in a banking branch and at the biometric station, and the varying suitability of the modality for the user [2,3].

The proposed process was designed for the engineered multimodal system, devised to incorporate any number of input biometric traits, currently with support for face image, 3d face geometry, voice, signature, hand vein, and gaze tracking. The verification can be performed based on any subset of these modalities, providing automatic adjustments to user capabilities. If a sample cannot be registered (e.g., voice distortion due to illness, signature illegibility due to hand injury, etc.), verification can be performed based on any other available modalities. The result is provided by a fusion of different decisions, each based on the analysis of a single trait. Our previous study shows that combining results from several biometric samples can provide a high probability of proper verification [3].

The multimodal biometric verification system can incorporate multiple sensing technologies to collect biological traits. Several popular approaches are used under a common notion of fusion designed to combine the raw data, processed information, or decisions in multimodal biometrics to increase the probability of correct classification. Data fusion in biometrics can be performed on four different levels [4,5]: (1) fusion of raw sensor data to create a high-resolution image or scan to be further processed by a single dedicated algorithm; (2) fusion of features from many modalities, based on concatenation or merging of feature vectors before performing the final decision, (3) fusion of comparison scores by joining results obtained from similarity or distance measurements for each input sample and template in the database; (4) fusion on the decision level, considering the combination of many binary decisions from separate modalities to make the final verification.

Multimodal biometric approaches, characterization of their strengths, weaknesses, typical processing phases, systems architectures, operation modes, algorithms, and levels and methods of fusion can be found in works of Oloyede and Hancke [6], Ross and Jain work [4,7], and Gudavalli et al. [8].

The notion of biometric identity is considered not only by engineers but also in philosophical discourse. A recent study by Kind [9] listed the most pressing ones: privacy, bias, security in data collection, and adding new metaphysical issues concerning matters of personal identity.

Regardless of the method, key quality criteria should be met, including robustness of the system, security, spoofing attacks protection, as recently characterized by Arora et al. [10] for the most novel approaches based on deep learning. Therefore, the correct data fusion procedure is proposed here and verified on synthetic and real biometric data to ensure proper handling of modalities that contribute to the final verification result.

Working with one of the largest European banks for several years, we have already conducted experiments on multimodal biometrics, organizing research experiments in 100 bank branches, involving almost 10,000 customers [3]. It allowed us to study the performance of individual biometric modalities in real banking branch conditions and to determine the opinions of bank advisors and customers about biometrics based on a survey. In the meantime, image and voice analysis methods have developed, and eye-tracking has become more widely used. Therefore, we implemented new face and voice recognition software in the current project, including artificial neural networks, and added mobile applications working on Android and iOS platforms. The bank has also decided to develop integrated workstations called “Biometric Island” to apply biometrics in bank branches more comfortably. However, the problem of combining the different modalities into a joint decision system had not been solved satisfactorily before. Therefore, the most important direction of our current research is biometric fusion. We wanted to answer how to fuse the results of biometric measurements to make a joint decision for effective confirmation of customer identity. Hence, we have developed a biometric fusion method and tested it, as is documented in this paper, especially in Section 5.

To show the context of this research, we briefly presented a biometric system built for this purpose. Still, the research part of the paper relates to how data fusion is solved when several techniques and modalities are used simultaneously in the identity authentication process.

The paper is constructed as follows. 

Section 2 shows the general design of a multimodal biometric verification system, Section 3 characterizes methods for data fusion, then the chosen comparison score fusion is presented (Section 4), with justification for the need of processing imprecise information and the usage of Dempster-Shafer Theory, commented in Section 4.2. Then, the process for scores fusion and its variants are presented in Section 4.3. Finally, the proposed methods are first validated based on synthetic data (Section 5) and the real biometric data collected by our system in its development phase (Section 6). The results show false non-match rates of all multimodal biometric modality pairs fused with six approaches (Section 7), following conclusions (Section 8).

## 2. Overview of Multimodal Biometric System

Section 2.1, Section 2.2, Section 2.3, Section 2.4 and Section 2.5 contain a detailed explanation of system parts: used sensors, data collection, algorithms for data processing and comparison of sample with stored patterns for verification, and finally, a fusion of all decisions.

### 2.1. Biometric Sensors

The multimodal biometric verification system consists of dedicated sensors for each modality. Due to their specifications, each has a different way of connection. At the same time, a software microservices approach allows for a common communication standard with the main software responsible for handling the cooperation and data processing. Table 1 lists the types of sensors and their connections.

For the common standard of data exchange, an MQTT was proposed. MQTT is a standard messaging protocol for the Internet of Things. It was designed as a lightweight messaging transport suitable for connecting remote devices with a small code footprint and minimal network bandwidth. A set of MQTT messages has been developed for each modality to allow cooperation with the central processing part and the frontend, responsible for user-related actions. These messages consider the specifics of each sensor type and modality, including sending configuration parameters, receiving data and processed information, triggering acquisition, etc. All sensors are operated using an MS Surface workstation with an additional screen connected for the intuitive and compact presentation of GUI, feedback information, guidelines, and verification results.

### 2.2. Data Collection Procedure

The operation of the system follows standard principles of biometric verification: a prior collection of mater template, i.e., a high-quality sample in controlled conditions, preceded by a semi-automatic verification using an ID card analyzed by the ID scanner or verified manually by a bank employee confirming the identity of a customer. The ID card was required only once when the client was enrolled for the first time, so their reference biometric samples were to be recorded and stored. New samples are collected and then compared by a dedicated algorithm with the reference template during the verification attempt. The specifics of data collection vary for each modality, but the result is saved in the sample database each time and used in the verification process.

The user receives visual feedback of successful sample acquisition and is guided to perform another one if required.

### 2.3. Identity Verification Procedure

Dedicated classifiers for each biometric trait have key characteristics measured in the following process. The respective verification algorithm was used for determining (1) scores for intra-class comparisons over all pairs of samples originating from one person, repeated for all persons, and (2) scores for inter-class comparisons for all pairs of identities in which the first sample was from a given person and the second was from all other individuals. This resulted in determining the minimum score for the case of mated comparison (genuine identity sample), and maximal score for the case of non-mated comparison (impostor identity sample), as well as the value of equal error rate (EER) for *j*-th modality, that is, the threshold for which the false non-match rate (FNMR) is equal to false match rate (FMR). The characteristics are again exploited in a fusion procedure to obtain base probabilities (Section 4.2.7).

Parameterized individual templates are used to verify the user’s identity. The verification is done by comparing the currently submitted templates with reference samples previously submitted by the user. The reference templates (in the form of parametric representation) are stored in the database.

**Figure 1:**
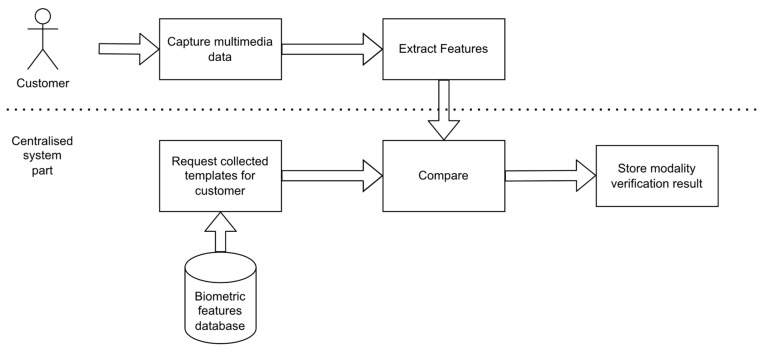
General steps for verification of single modality.

Figure 1 presents the process of verification service consisting of acquiring templates for verification, obtaining biometric templates from the database for comparison, using developed algorithms for features extraction and features comparison, and optionally presenting the result of individual verifications.

### 2.4. System Architecture

**Figure 2:**
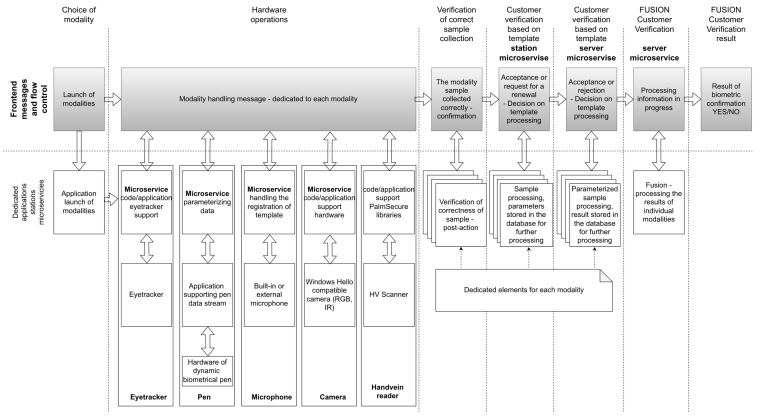
Multimodal biometric system architecture.

The architecture of the engineered experimental system was conceived to implement the described process approach as a biometric infrastructure installed in real banking branches. The architecture diagram in Figure 2 shows the process realization with sensors dedicated to acquiring multimedia data used in particular types of biometrics. The samples collected in a given order by dedicated hardware are processed individually by biometric verification algorithms, resulting in quality confirmation and distance (or similarity) measurements. These results are processed by the fusion module, finally providing identity verification (Figure 2).

The architecture was prepared using a microservices approach focused on the possibility of running in the cloud, outside of the client device, without the need to transmit reference templates for comparison. The prototype consists of microservices supporting individual modalities (the part installed on the station and the central part) and handling the fusion. The MQTT protocol was chosen as the standard for communication, supporting control events, information events, and transmission of parameterized encrypted samples.

The architecture of the software modules is highly distributed. For example, two microservices, one for calculating biometric fusion and the other for handling gaze tracking acquisition and processing, are running containerized on a Linux server in a cloud virtual machine. Other key parts dedicated to each modality are located on a cloud-based virtual machine running MS Windows Server Datacenter. Additionally, on a separate virtual machine are running:

- Apache server supporting two versions of the frontend application used on the MS Surface screen and monitor screen, communicating with each other using the indicated protocol,
- RabbitMQ communication server—supporting communication between solution components,
- MongoDB database with a dedicated microservice to handle all interactions with the database.

This modular and distributed solution was verified to be highly scalable and adaptable to other environments, operating in a protected architecture and virtual networks, fitting the critical security requirements defined by GDPR [11] and ISO/IEC 24745 Norm [12].

**Figure 3:**
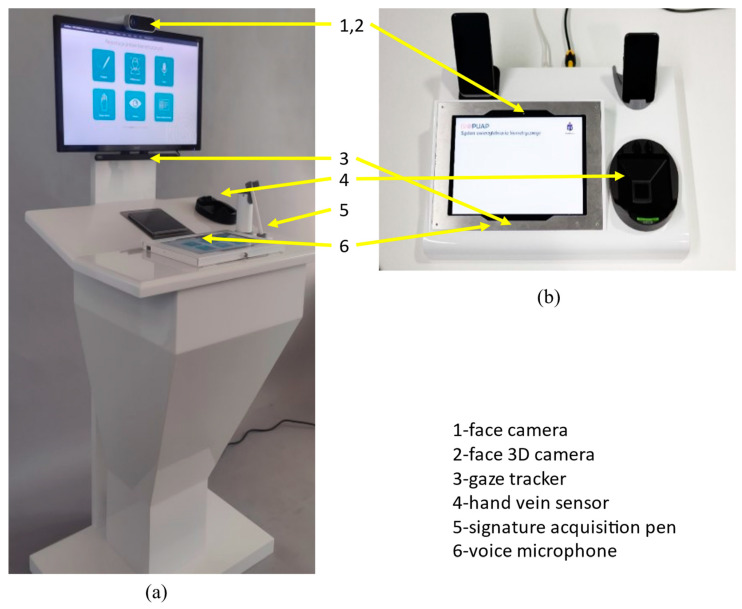
Biometric stands: for standing and sitting clients. Biometric devices are visible: (**a**) face camera, face 3D camera, a voice microphone, signature acquisition pen, hand vein sensor, gaze tracker, (**b**) hand vein sensor, gaze tracker, face camera, face3D camera, voice microphone.

Two types of biometric stands were developed, one to be operated in a standing position and one while sitting at the desk (Figure 3).

### 2.5. Decision Fusion

**Figure 4:**
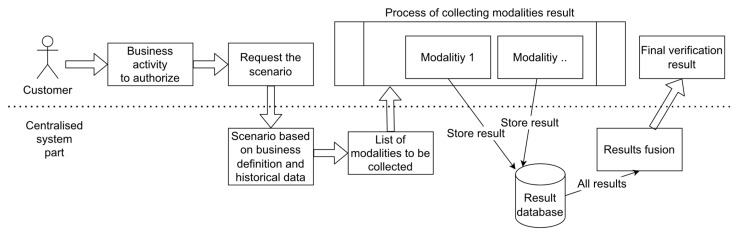
Block diagram of the fusion process.

The biometric authorization of conducted banking activity (electronically signing a document, confirming a banking transaction or other) is based on the fusion of separate results from many modalities. The process is presented below (Figure 4). In the first step, the fusion module prepares a scenario, i.e., a list of modalities that the client should verify. The scenario dedicated for a given activity and a given client depends on (1) the level of criticality of the banking activity, (2) the list of templates available for the client, (3) and the history of previous verifications. After the fusion module presents the list of modalities, the frontend manages the samples acquisition. After completing the collection of individual results, the algorithm of the fusion module calculates the final value based on the collected partial probabilities from the modalities, and the verification result is presented to the client in the form of a message.

## 3. Multimodal Biometric Fusion Methods

Fusion levels are briefly characterized below, and justification is provided on the choice of comparison scores fusion and Dempster-Shafer Theory for our multimodal biometric verification system.

### 3.1. Feature Level Fusion

Fusion of features from many modalities first requires collecting and pre-processing various traits from the user, then extracting distinct features expected to help distinguish individuals. Separate vectors or maps of features are merged by concatenation, next used in the final classifier. Compared with other fusion levels, the feature level is generally considered [13,14] to be the most effective. The fused features allow for efficient distinction in highly-dimensional space between the non-mated (impostor) and mated (genuine) samples, similar to stored biometric templates. Thus, feature level fusion provides more distinctive information than many uni-modal classifiers. Each produces a single real value of trait sample similarity measure, finally fused and analyzed in low-dimensional decision space.

Haghighat et al. [13] used discriminant correlation analysis (DCA) at a feature level, maximizing the pairwise correlations and reducing between-class correlations. Their method was verified on different databases with three and six modalities. Shekhar et al. [14] proposed a feature level fusion by the multimodal sparse representation of training data linear combination with the coupling of information among biometric modalities of a subject. Hezil and Boukrouche [15] proposed feature-level fusion of ear and palmprint data, showing an increase in recognition rates compared with single-modal biometrics.

Fusion of features improves classification accuracy but becomes computationally demanding as the dimensionality of fused data increases significantly. Moreover, the fusion of features is not applicable when the set of input modalities is expected to change due to failures, lack of dedicated devices, or user inability to use a given modality. This limitation is addressed in our work, where we assume that any number of supported modalities can be used in decision-making.

### 3.2. Comparison Score Level Fusion

Fusion of comparison scores (previously in literature entitled “matching scores”, later changed to “comparison score”, following the ISO Norm [16,17]) is performed by merging results obtained from many modalities, first calculating similarity or distance measurements for the input sample and template from the database. This method requires the knowledge of each trait biometric algorithm accuracy metrics (e.g., false non-match and false match rates) and proper weighting or normalization before the fusion [18,19]. Usually, functions such as t-norms are used to merge the input scores, using min, max, or fuzzy logic combinations of results.

Extensive evaluation of score level combination approaches using t-norms is presented by Hanmandlu et al. [20], addressing levels of uncertainty for different modalities. In addition, FL membership functions are used to model genuine, and impostor scores and fuzzy integration is employed to obtain the result. Finally, Ross and Jain addressed the fusion comparison score level on the face, fingerprint, and hand geometry multimodal biometric system [19].

The comparison score level fusion approach is simple and can be used for any number of input modalities. Each modality is first processed independently. Then, they can have different score ranges, distances, or similarity, normalized before the final fusion. Moreover, the additional calculations related to fusion are computationally efficient and introduce a significant increase in classification accuracy. Besides already mentioned t-norms and FL, another interesting method for fusion on the comparison score level is the Dempster–Shafer Theory, discussed in Section 3.4 and characterized in detail in Section 4.2.

### 3.3. Decision Level Fusion

The highest level the fusion can be performed on is the decision level. It requires a combination of many binary decisions from separate modalities to perform the final verification. First, each modality should be processed independently, and the input sample classified as positive or negative (mated or non-mated, genuine or impostor), employing dedicated methods (e.g., distance-based). Then, the binary classification results are fused employing logic operations, e.g., min, max, min-max operators. Decision-level fusion is often enhanced with fuzzy logic (FL) reasoning approaches. Abdolahi et al. [21] employed decision level fusion with results weighted in the final decision. The method is extended with fuzzy logic (FL) combination. Benaliouche and Touahria [22] examine iris and fingerprint fusion at the decision level and comparison score level, employing normalization of scores and then FL comparison scores combination, arguing that the FL decision fusion mimics the human reasoning.

The approach is computationally efficient, but its main drawback is reducing detailed information of lower levels (e.g., features, scores) and relying only on processed results of final single-modal decisions.

### 3.4. Dempster–Shafer Theory in Data Fusion

Dempster–Shafer Theory (DST) is widely used in many domains requiring a combination of many pieces of evidence or results to formulate a significant final decision. Mohandes and Deriche [23] employed DST for biometrics of hand motion, fusing information from a cyber glove and a hand motion tracking system at a decision level to recognize signs with a significant increase of accuracy.

Arif et al. [24] presented the applicability of DST in biometric verification based on signature and hand shape, showing a significant increase in accuracy and decrease in false non-match and false match rates. Singh et al. [25] employed DST to fuse comparison scores of various fingerprint-based verification approaches, including analysis of scores of minutiae, ridges, multi-frequency analysis of prints by the bank of filters, and pores characteristics, in result increasing key performance metrics of this multimodal verification. Cuzzocrea and Mumolo [26] employed a cascaded approach: fused fingerprints and voice with weighted sum and fuzzy logic, both treated as two decisions that were finally merged applying DST.

The main strength of the DST is the ability to express and process imprecision accordingly; therefore, it was used in our system as is characterized (Section 4.2) and examined in detail (Section 5).

## 4. Implementation of Comparison Score Fusion

A dedicated algorithm for processing the given modality calculates a difference or similarity between the input sample and the pattern features of the previously enrolled identity. Details of the algorithms were described earlier in our work [2,3,27] and are briefly characterized below.

Generally, the comparison result can be expressed as a Euclidean distance in n-dimensional feature space, measured between the input and one closest pattern or the center of a cluster of all stored patterns. The smaller the distance, possibly 0, the higher the similarity between the user and the declared identity read from the database. Selected algorithms also calculate the score for similarity, with a high score interpreted as a high similarity. All methods operate in feature spaces obtained by transforming the raw features or biometric signals to a representation more suitable for classification. In our work following approaches are implemented [28]:

- Face modality—the minimal distance in face keypoints descriptors 768 features space, composed of Histogram of Oriented Gradients and Local Binary Pattern features of 77 characteristic face landmarks, transformed with the Linear Discriminant Analysis [27,29],
- Voice modality—the maximum similarity of the speaker identity in the mel-cepstral frequency coefficients decision space statistically modeled with Gaussian Mixtures and Universal Background Models [3,30],
- Signature—accelerometer and a gyroscope signals processed with the triplet loss method, involving training a neural network to learn a new latent space representation, most suitable for maximization of the distance between signatures from different persons and minimization of the distance between signatures of the same person [31],
- 3D face image—the minimal distance between parameterized 3d meshes [32],
- Gaze tracking—the minimal distance in descriptor space, including statistical features of registered rapid eye movement speed (saccades), average, maximal, standard deviation, acceleration, length, distance, etc.
- Hand vein pattern—binary decision in a commercially available proprietary hardware unit by Fujitsu Identity Management and PalmSecure [3,33].

In any detection application, including the biometric verification with binary decision considered in our work, the problem arises with the outcome interpretation. Therefore, a threshold is required for converting the real value expressing the distance or similarity into the final binary detection. This threshold is set considering the false match ratio and false non-match ratio. The threshold should be optimal for minimal errors, the false match being the most harmful in the case of biometric identity verification applications. Some objective characteristics could be calculated to express the accuracy of the identity verification algorithm discussed below.

### 4.1. Characterization of Biometric Algorithms 

**Figure 5:**
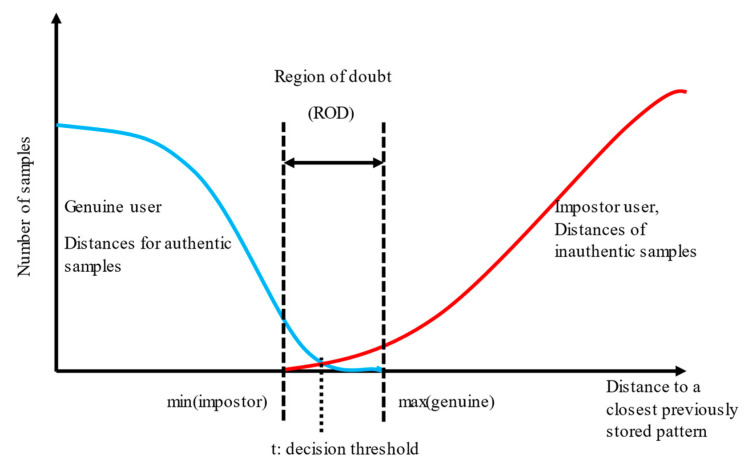
A model of decision space: a binary decision can be made depending on the distance between the input sample and the closest pattern. The minimal distances for impostor and maximal for genuine sample denote the width of the region of doubt. Modelled distributions shown in color: blue line—results for genuine samples, red line—results for impostor samples.

Let us consider only the case of an algorithm calculating “distance” (opposite to the “score”, for which the following observations would stay inversely true). The maximal distance from the previously stored patterns should be as small as possible for all correct identity samples. The highest among the measured distances characterizes the worst case for the mated comparison (genuine user). On the other hand, the minimal distance for non-mated comparison (an impostor), compared with all stored patterns, should be as high as possible, and the smallest one is the worst-case scenario. Both values, here informally named min(impostor) and max(genuine), denote boundaries of the region of a doubt (ROD), where the distance value for unknown input sample would not allow precise classification as a genuine or impostor (Figure 5). Therefore, ROD should be as small as possible, optimally none.

Any result with the distance value lying in ROD supports both hypotheses partially: the class could be genuine, or, at the same time, the class could be an impostor. Results below min (impostor) fully support the hypothesis that the class is genuine, and a value above max(genuine) supports the impostor class. 

Probabilities can be calculated, resulting in estimated values of p(genuine), p(both), p(impostor) that would be usable in an actual application. A method is required to efficiently deal with such data and combine many imprecise and unreliable results from many detectors (i.e., many modalities). 

The thresholding of the distance and performing binary classification returns a binary decision. However, details about the underlying calculations, e.g., maximal genuine distance minimal impostor distance discussed above, are lost. Therefore, we propose a method processing all intermediate results and considering real values of probabilities available in each classifier before the final discrete decision is made. Our work addresses these issues by exploiting mass functions from Dempster-Shafer Theory [34], following modifications [35], and combining results from multimodal detectors.

### 4.2. Dempster-Shafer Theory Application in Biometric Verification

Theory of evidence, introduced by Artur Dempster and then popularized by Glen Shafer [34] (DST—Dempster-Shafer Theory), was created to introduce a method for dealing with imprecision and uncertainty. Contrary to the classic probability, the knowledge about facts is defined and processed differently from uncertain information. Therefore, imprecise biometric identity verification can be done in a DST framework instead of calculating Bayesian probability.

#### 4.2.1. Imprecision vs. Uncertainty

The lack of knowledge under some available data, observations, or evidence can be used to express two things: lack of precision (can be measured by observing characteristics of a sensor and classifier) and uncertainty (unmeasurable). The knowledge about the subject of interest (e.g., biometric identity) could be extended by incorporating many sensors and multiple measurements, thus positively or negatively changing the precision and uncertainty.

On the one hand, uncertainty occurs when it is impossible to determine whether the sentence is true or false. Typically, these are measured by probability functions in the Bayesian approach. This approach is beneficial, widely understood, applied, and used to make conclusions, although not appropriate for all cases.

On the other hand, the lack of precision is when the statement does not contain precise values, such as an expression of a biometric result by binary thresholding or any other value definition involving ranges or even fuzzy sets.

The problems were addressed in numerous works and are widely known in the literature [34]. The aspect of imprecision was often considered in applications such as biometrics [24,25,26,36], and some examples were provided in Section 3.

The probability-based approach to imprecision is limited and does not match the human way of thinking and solving practical problems. For example, behavior and decision-making are not correlated with a measurable probability [37]. Moreover, the probabilistic methods do not provide a framework for expressing and dealing with all types of knowledge.

DST is based on probability but extends the approach with a lack of knowledge by introducing a belief model. 

#### 4.2.2. Belief Model in DST

The notion of degree of belief was introduced in the DST to model human confidence and certainty about observed data and to allow machine calculations on the data that a person typically handles, e.g., a bank employee verifying client identity by subjectively rating the similarity between the photo in the ID and the client’s face. The belief is quantified by base probabilities, defined below, allowing imprecise information processing.

Moreover, belief value is related to the following domains: cognitive—would aid in understanding psychological process underlying the decision-making; normative—would help calculate and express rules for critical applications, decision-makers can understand that; pragmatic—would be helpful to in expert systems to simulate natural processes of decision-making whenever the value of belief is crucial [38].

#### 4.2.3. Mass and Base Probability

The abovementioned base probabilities are called “masses” in DST [34] and express the known amount of probability assigned to any given subset of all facts. The mass can be assigned to single elements, sets of elements, or not assigned. Some elements can have unknown mass as well. The following definitions were introduced by Dempster and Shafer [34]:

- Ω—universal set of all facts, e.g., Ω = {a, b}, later also denoted as *e* (“either”)
- 2^Ω^—power set of all possible subsets of Ω, e.g., 2^Ω^ = {ø, {a}, {b}, {a, b}}
- m( )—mass function, the base probability function for any of elements in 2^Ω^, following three properties (1):
  m: 2^Ω^ → [0, 1], m(ø) = 0, Σ_A__∈2Ω_ m(A) = 1. (1)

Substituting A with Ω in (1) would result in m(Ω) = 1, because: m(ø) + m({a}) + m({b}) + m({a,b}) = 1. The element m({a,b}) allows for modelling the lack of knowledge, as it can have any mass value between 0 and 1, decreasing the individual masses of m(a) and m(b), as it is allowed for any mass to be 0. 

Let’s say: m(ø) = 0, m({a}) = 0, m({b}) = 0.4, m({a, b, c}) = 0.6. This case is interpreted as the lack of knowledge about probabilities of a, b, and c, but some mass assignment is made to support “b” and “any of a or b or c”.

Mass of any subset A ∈ 2^Ω^, m(A) is interpreted as a measure of all valid and available evidence that the observed fact is in the A as a whole, but without the assumptions about particular A’s subsets (m(A) considers only whole A and cannot yield any conclusions about subsets). If A = {{b}, {c}} and m(A) is known, the masses m(b) and m(c) would still be unknown.

#### 4.2.4. Probability in the DST Belief Model

The classical probability is derived from the mass values and expressed by lower and upper limits [34]. The p(A) is expected to be in the range (2):

Bel(A) ≤ p(A) ≤ Pl(A)(2)

where Bel(A) is the belief for A and Pl(A) is the plausibility of A, defined as follows (3):

Bel(A) = Σ _B__⊆A_ m(B),
Pl(A) = Σ _(B__∩A)__≠∅_ m(B). (3)

Belief for any set is a sum of masses of its all subsets, and belief for a single element {a} is equal to its mass: Bel({a}) = m({a}).

The relation between plausibility and belief is as follows: Pl(A) = 1 − Bel(¬A), and the plausibility is interpreted as the strength of evidence not invalidating A (1 minus the belief of evidence invalidating A).

The range between upper and lower probability limits would change due to providing more knowledge. The more masses are available, the more precise values of belief and plausibility would be, starting with Bel = 0, Pl = 1 and 0 < p(A) < 1 for the lack of any knowledge, and ending with a single precise value of the true probability Bel(A) = p(A) = Pl(A).

More on that matter and precise analysis can be found in original work from Dempster and Shafer [34], as well as other publications [39,40,41,42], including a critique by Zadeh [43], Haenni’s defence [41], modifications by Yager [42], Smets [36], and Denoeux [39].

#### 4.2.5. Probability Estimation Methods

As shown above (2), the masses can be used to calculate lower and upper limits of probability (belief and plausibility, respectively), allowing imprecision to be expressed. However, the two-valued probability estimation is not directly applicable to the decision systems. Therefore, some work proposed the conversion of masses, belief, and plausibility to probabilities. For example, a pignistic probability (latin: pignus—bet, wager) was introduced by Smets [36] to provide a simple way of assessing “rational” probability. The notion originates in the observation that a rational person with insufficient knowledge and no previous assumptions about probabilities will assign equal chance to all options.

One can observe that for a case where: m(a) = 0.2, m(b) = 0.2, m(c) = 0.3, m({a, b, c}) = 0.3 the last value is related to a mass distributed to “a or b or c” in unknown proportions, but the total is known to be 0.3. Calculating the pignistic probabilities p_pign_() involves assuming the proportions are equal, dividing the masses by the cardinality of the sets, and then combining masses accordingly: p_pign_(a) = m(a) + 0.33·m({a, b, c}), p_pign_(b) = m(b) + 0.33·m({a, b, c}), p_pign_(c) = m(c) + 0.33·m({a, b, c}), finally resulting in probabilities: p_pign_(a) = 0.3, p_pign_(b) = 0.3, p_pign_(c) = 0.4. One can observe that Bel(a) = 0.2, Pl(a) = 0.2+0.3 = 0.5, and the pignistic probability is usually close to the mean of belief and plausibility, but that depends on the mass assigned to the {a, b, c}.

Another method for estimation of the true probability is taking into account the plausibility values [44], i.e., the upper limit of the probability. Let’s consider the same example: m(a) = 0.2, m(b) = 0.2, m(c) = 0.3, m({a, b, c}) = 0.3. Then, following the definition (3): Pl(a) = m(a)+ m({a, b, c}) = 0.2 + 0.3 = 0.5, Pl(b) = m(b) + m({a, b, c}) = 0.2 + 0.3 = 0.5, Pl(c) = m(c) + m({a, b, c}) = 0.3 + 0.3 = 0.6.

It should be observed here that the sum of plausibilities can be higher than 1. Therefore, the so-called relative probability p_rel_() will be obtained after a normalization:

p_rel_(a) = Pl(a)/Σ_x__∈Ω_ Pl(x) (4)

Thus, relative probabilities in this case are: p_rel_(a) = 0.3125, p_rel_(b) = 0.3125, p_rel_(c) = 0.375.

#### 4.2.6. Masses and Probabilities for a Binary User Identity Verification

Let us say there are only two possible values of decisions in the binary biometric system: *g* for genuine and *i* for impostor, *e* for either or undetermined (*e* is introduced only for a shorter notation: {*g*,*i*} = *e* and *e =* Ω). Based on masse values, the following can be measured and treated as the lower and upper limits of probabilities of *g*, *i,* and *e* (Table 2).

In the Table 2 the relative probability is based on (4), where the sum of all plausibilities is:

Σ _x__∈({g,i})_ Pl(*x*) = Pl(*g*) + Pl(*i*) = m(*g*) + m(*e*) + m(*i*) + m(*e*) = 1 + m(*e*),

because the sum of all masses is 1. Moreover:

p_p_rel_(*g*) = Pl(g)/(1 + m(*e*)) = (m(*g*) + m(*e*))/(1 + m(*e*)) = (m(*g*) + m(*e*) + m(*i*) − m(*i*))/(1 + m(*e*)) = (1 − m(*i*))/(1 + m(*e*)),

and:

p_p_rel_(*i*) = (1 − m(*g*))/(1 + m(*e*)).

Then:

p_p_rel_(*g*) + p_p_rel_(*i*) = (1 − m(*i*) + 1 − m(*g*))/(1 + m(*e*)) = (2 − m(*i*) − m(*g*))/(1 + m(*e*)) = (1 + m(*g*) + m(*i*) + m(*e*) − m(*i*) − m(*g*))/(1 + m(*e*)) = (1 + m(*e*))/(1 + m(*e*)) = 1,

and both pignistic and relative probabilities of *g* and *i* properly sum to 1. Finally, one can observe that for the binary case, the pignistic probabilities are always the mean of Bel( ) and Pl( ), and the relative probability is the lower, the higher is the m(*e*) in the denominator, expressing the lack of knowledge.

#### 4.2.7. Conversion of Classifier Score to Mass Value

Assuming that the characteristics of the detector (the maximal score for impostor and minimal score for genuine identity, or maximal distance for genuine and minimal for impostor) is known, the following procedure can calculate the base probabilities we adapted in our work after Mezai et al. [45].

The conversion is conducted based on the value of the score for the *j*-th classifier for the *k*-th input sample. First, a distinction is made based on the position of score value respective to the boundaries of ROD area. When the score is not in the ROD, and is high enough to surpass the max(impostor) value:

if(score_jk_ > max_imp_):

m_jk_(*g*) = f_j_(score_jk_),

m_jk_(*i*) = 0,

m_jk_(Ω) = 1 − f_j_(score_jk_).

When the score is not in ROD, and is lower than the min(genuine) value:

else if(score_jk_ < min_gen_):

m_jk_(*i*) = 1 − f_j_(score_jk_),

m_jk_(*g*) = 0,

m_jk_(Ω) = f_j_(score_jk_).

When the score is in ROD, but larger than the threshold:

else if(score_jk_ > t_j_):

m_jk_(Ω) = f_j_(score_jk_),

m_jk_(*g*) = 1 − f_j_(score_jk_),

m_jk_(*i*) = 0.

When the score is in ROD, but larger than the threshold:

else if(score_jk_ < t_j_):

m_jk_(Ω) = 1 − f_j_(score_jk_),

m_jk_(*i*) = f_j_(score_jk_),

m_jk_(*g*) = 0.

Above, the f_i_( ) is a function scaling the score of *j*-th detector to a range [0, 1], that for high enough scores (or for low enough distances) should be 1, e.g., sigmoid:

(5) $$f_{j}\left(score_{jk}\right)={\left(1+e^{-r\cdot \frac{\left(score_{jk}-t\right)}{max\left(score_{j}-t\right)}}\right)}^{-1}$$

Introduced notation:

*j*—the *j*-th modality detector
*k*—the *k*-th examined sample
Ω—the set of probable facts Ω = {*g*, *i*}, further denoted also as *e* (“either”)
m()—base probability, mass
*t_j_*—threshold for which the f_j_(score_jk_) will be 0.5.
*r*—curve parameter, the speed of saturation of the f_j_( ), depends on the design decision about the score value where the mass should be large enough, e.g., surpass 0.9.

For all cases above, one of the elements in Ω has a mass equal to 0, and m_jk_(Ω) expresses the lack of knowledge, which will be processed accordingly to the selected probability estimation method. 

**Figure 6:**
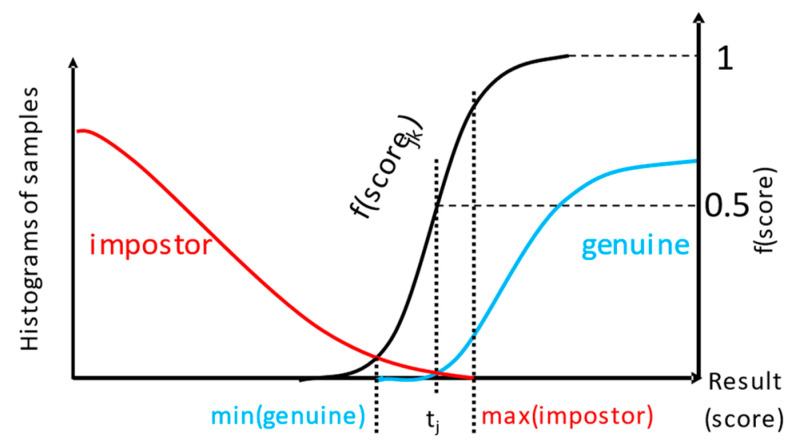
The sigmoid f( ) used for calculating mass, depending on the score for input biometric sample.

The illustration of the principle for f( ) design implemented in our work is presented in Figure 6. Higher values of curve parameter *r* would result in the steeper function, reaching 0 faster for scores lower than *t*_j_, and quickly saturating at 1 for scores higher than max(impostor), which have a very high probability of being the result of a genuine input sample.

### 4.3. Fusion of Decisions in Multimodal Binary Classification 

In multimodal biometrics, numerous sensors and algorithms are used, dedicated to acquiring and processing many different traits, and each calculating the masses of facts based on different samples. Let us consider a case of two classifiers, returning base probabilities: m_1_(*g*) = 0.95, m_2_(*g*) = 0.9. These masses can be fused in many possible ways to express the total probability that the user’s identity is genuine.

The simplest way to obtain final decision is to assume all other masses can be neglected, and to calculate joint probability, that both decisions are *g* at the same time: m_1_(*g*)·m_2_(*g*) = 0.95·0.9 = 0.855, then probability that both detections are false: (1 − m_1_(*g*))(1 − m_2_(*g*)) = (1 − 0.95)(1 − 0.9) = 0.05·0.1 = 0.005, and finally the probability that at least one detection is true: 1 − 0.005 = 0.995. The last value is the plausibility that can be used to make a final decision. It should be observed that the fused probability is higher than its individual parts, and the process allowed for an increase in the system accuracy.

#### 4.3.1. Dempster’s Fusion of Evidence

The fusion of results should increase the final probability the input identity is genuine. Even if specific base probabilities are not high, the operation of many detectors in concert should increase the accuracy of the system. 

Dempster proposed merging masses in the following way [34]:

(6) $$m_{3}\left(A\right)=m_{1}\left(A\right)⊕m_{2}\left(A\right)=\frac{\sum_{B\cap C=A}m_{1}\left(B\right)m_{2}\left(C\right)}{1-\sum_{B\cap C=\emptyset }m_{1}\left(B\right)m_{2}\left(C\right)}$$

The denominator is 1 − K, where K is interpreted as a measure of conflict between evidence. The outcome mass m_3_ is high for cases of agreement between evidence. If many instances of evidence in *A* are conflicted, then the mass of the only evidence not conflicted is significantly increased. For large K, the nominator is going to 0, then the result m_3_ goes to 1.

Let us consider masses measured for three biometric identities: id_1_, id_2_, id_3_. One can observe that when evidence is highly conflicted: m_1_(id_1_) = 0, but m_2_(id_1_) = 0.9, and m_1_(id_2_) = 0.9, but m_2_(id_2_) = 0, and conflicted evidence m_1_(id_3_) = 0.1, m_2_(id_3_) = 0.1 does not exist, the resulting mass for this evidence would be unintuitively high: m_3_(id_3_) = 1.

In this regard, the Dempster rule of combination is often criticized and compared to the logical AND operator: (m_1_(id_1_) = false) AND (m_2_(id_1_) = true) THEN (m_3_(id_1_) = false). Therefore, the rule should be applied only for not conflicting evidence from two systems or detectors.

#### 4.3.2. Modified Combination of Evidence

It was proposed by Yager [42,46] to calculate the mass of conflicting evidence differently, assuming that mass from conflict K should be assigned to the empty set, thus expressing lack of knowledge, and masses for useful evidence will only count agreeing cases:

(7) $$m_{3}\left(A\right)=m_{1}\left(A\right)⊕m_{2}\left(A\right)=\sum_{B\cap C=A}m_{1}\left(B\right)m_{2}\left(C\right)$$

Other modifications, instead of beliefs, only consider plausibilities, thus reducing the mass of uncertain evidence [44]. When the knowledge is complete and masses of single elements sum to 1, the result of modified DS and original DS are the same. 

## 5. Fusion Methods Evaluation

In the beginning, a banking client case was modeled to examine fusion methods, where input score_1_ and score_2_ from two different modalities are synthetically generated, covering all accepted ranges of values, starting with scores equal to 0, then increasing with a step of 0.1 up to the maximal value of 1.0. This resulted in 11 values for score_1_, 11 values for score_2_, thus 121 combinations in total.

First, each given score value is converted to masses of *g*, *i,* and *e*, following the procedure described above (5) (Figure 6). As a result, masses from the first modality: m_1_(*g*), m_1_(*i*), m_1_(*e*), and the second modality m_2_(*g*), m_2_(*i*), m_2_(*e*) are obtained subsequently processed by the fusion module. Next, the three methods are employed to calculate m_3_(*g*) as a result of fusing respective masses of m_1_( ) and m_2_( ). The mass is finally converted into probability, using pignistic and comparative approaches.

For visualization purposes, the score_1_ and score_2_ values are normalized, to the range [0, 1], with a 0.5 value for the decision threshold. The score value 0 means the identity is false, and the impostor’s mass m(*i*) is 1. Then, for the score in a range (0, 0.5], a gradual decrease in mass of m(*i*) and increase in mass of m(*e*) is observed, as the amount of clear evidence for impostor class decreases. However, there is still not enough knowledge to support genuine identity. Therefore, the mass of either *g* or *i* increases to m(*e*) = 1. A subsequent increase in the score value from 0.5 to 1 results in an increase in mass of genuine class m(*g*) and a decrease of m(*e*) to zero, whereas the sum of masses is always equal to 1.

**Figure 7:**
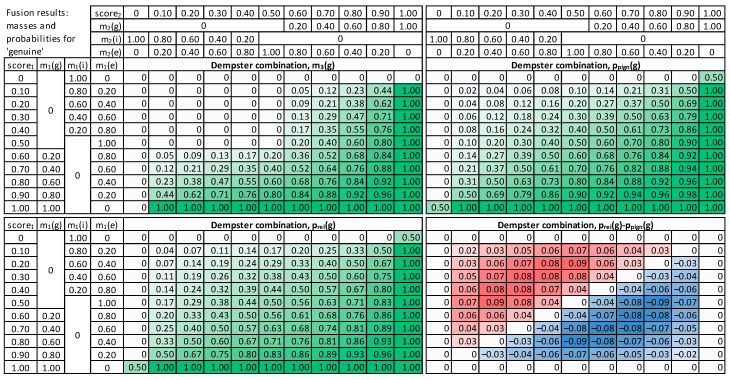
Results of Dempster combination, mass m_3_(*g*), pignistic probability p_pign_(*g*), relative probability p_rel_(*g*), and a difference between these probabilities.

For the Dempster combination method (Figure 7), it is visible that when any input mass of *g* is zero, the result is also 0 (upper left quadrant of Dempster combination table). It can be observed that probability value increases with the increase n any of the score_1_ or score_2_. The relative probability is usually higher than the pignistic approach for the “upper half”, i.e., cases when the sum of score_1_ and score_2_ is low. When the false acceptance is crucial for practical applications, the Dempster combination with pignistic probability is better than the relative probability because it results in lower probabilities for low input scores.

**Figure 8:**
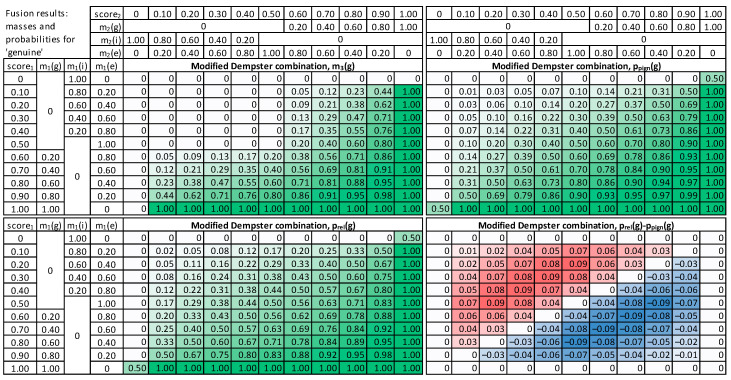
Results of Modified Dempster combination, mass m_3_(*g*), pignistic probability p_pign_(*g*), relative probability p_rel_(*g*), and a difference between these probabilities.

The modified Dempster approach (Figure 8) is similar to Dempster, but with a faster increase towards high masses and probability values, in consequence of score increase, and smaller values for low scores. Comparing the two methods for calculating probabilities, it is visible that the relative probability values are higher than pignistic ones for the cases when both input scores are low. Therefore, the Modified Dempster combination with pignistic probability would be favorable for practical applications when a false match case is crucial.

**Figure 9:**
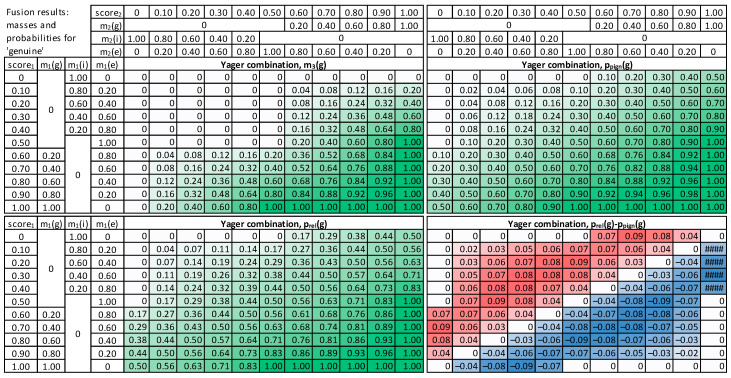
Results of Yager combination method, mass m_3_(*g*), pignistic probability p_pign_(*g*), relative probability p_rel_(*g*), and a difference between these probabilities.

The Yager fusion method is expected to properly handle cases of significant conflict between two combined masses (Figure 9). It can be observed that the m_3_(*g*) for low score_1_ and high score_2_ is always very low, contrary to both Dempster and Modified Dempster shown above (last column and last row in “Yager combination, m_3_(*g*)” table).

**Figure 10:**
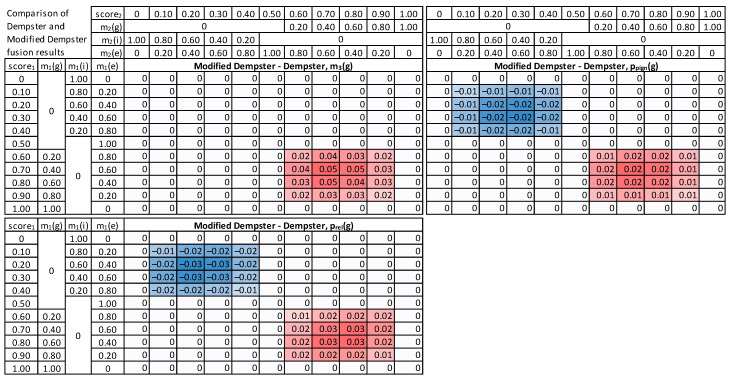
Differences between Modified Dempster and the original Dempster method: masses, pignistic probabilities, and relative probabilities.

**Figure 11:**
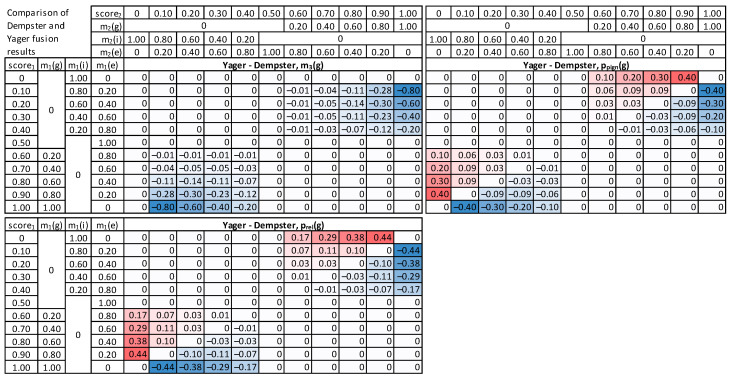
Differences between Yager combination method and the original Dempster method: masses, pignistic probabilities, and relative probabilities.

The fusion results were further examined, and a direct comparison of the original Dempster approach with Modified and Yager versions was made (Figure 10 and Figure 11). The modified approach returns higher masses and higher probabilities for high scores. The Yager’s method (Figure 11) produces significantly lower masses for high conflict of scores (up to 0.8) than the Dempster method. As a result, the probabilities calculated based on Yager masses are the lowest among all methods in case of conflicting inputs.

The evaluation showed strong and weak points of the methods considered in the study. Designing a multimodal biometric identity verification system should consider the characteristics of the fusion algorithm and the application-specific behavior of the system in cases of conflicting inputs. The appropriate action should be performed whenever the input sample of one modality invalidates the final verification.

The final probability value obtained from the fusion process should be compared against the defined verification threshold, and the identity should be treated as genuine only in cases of high probability, for example, surpassing 0.9 thresholds or higher. In the evaluation of the method above, it can be observed that such cases usually occur when both input masses are higher than 0.8.

## 6. Fusion Experiments on Multimodal Biometrics Dataset

The multimodal biometric verification system was used in the subsequent course of research to collect numerous enrolment and verification samples. The results from individual modalities were fused to obtain the combined value of the probability of genuine identity. 

### 6.1. Biometric Database

The database we created included biometric samples from 197 individuals, 4744 samples in total, with six different modalities registered. Due to the ongoing development, some biometric traits were used more frequently than others (Table 3).

Among the 197 users, there were cases of registering only a single modality or many modalities. There were 980 registration sessions of different lengths, as presented in Table 4.

The fusion was performed for any two input modalities acquired in a single session from one individual. There were 256 pairs, with more registrations for Voice and Face and Signature and Voice (Table 5).

### 6.2. Experiment Design

The key characteristics described in Section 4.2.7 were measured for all modalities employing all collected biometric samples. First, the score denoting min(genuine) was determined by applying the respective verification algorithm and comparing all pairs of samples originating from one person, repeated for all 197 persons (scores for intra-class comparisons). The max(impostor) was calculated by comparing all pairs. The first sample was from a given person, and the second was from all other individuals (scores for inter-class comparisons). This resulted in finding the range of doubt (ROD). Finally, the threshold t_j_ was determined as the value minimizing the EER—equal error rate for *j*-th modality, that is, the threshold for which the FNMR=FMR, false non-match rate is equal to false match rate.

The procedure allowed us to decide on the score-to-mass conversion characteristic f_j_() for each *j*-th modality. During the system operation, the input samples were analyzed, and the base probabilities were reported as masses: m_jk_(*g*), m_jk_(*i*) and m_jk_(*e*). Next, the fusion was conducted with the three studied methods introduced in Section 4.3. 

The study goal was to verify approaches for combining the masses and calculating the final probability of the genuine class: three variants of combination and two variants for probability calculation were examined.

All the sessions with less than two modalities were rejected. Then all pairs of modalities were fused using three approaches: (1) Dempster Combination, (2) Modified Dempster Combination, and (3) Yager’s Rule. Next, the resulting masses were converted into (1) pignistic and (2) relative probabilities. Finally, the probability in each case was compared with the determined decision threshold, adjusted in the range 0.6 to 1.0, to measure the false non-match rates and create FNMR characteristics in each case. 

## 7. Results

**Figure 12:**
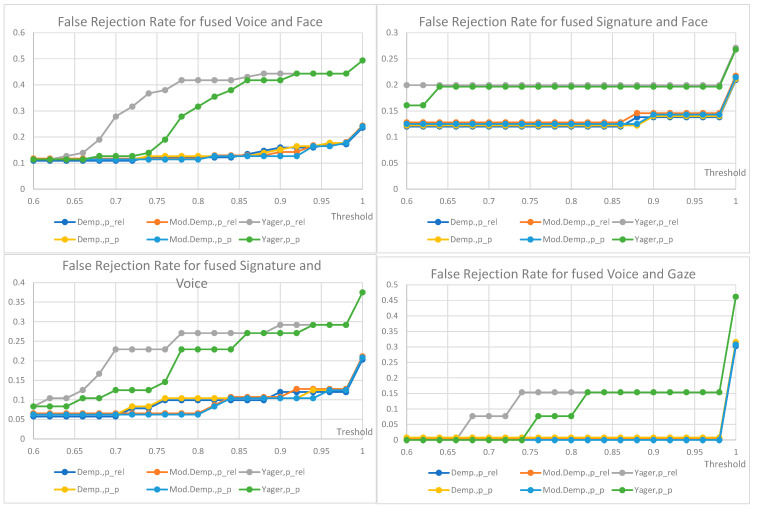
FRR for the fusion of voice and face (79 pairs), FRR for the fusion of signature and face (56 pairs), FRR for the fusion of signature and voice (48 pairs), FRR for the fusion of voice and gaze (13 pairs).

**Figure 13:**
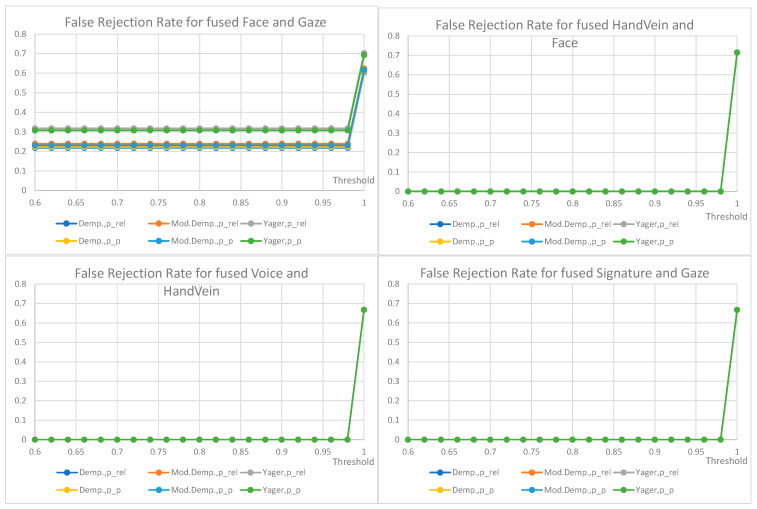
FRR for the fusion of face and gaze (13 pairs), FRR for the fusion of hand vein and face (7 pairs), FRR for the fusion of voice and hand vein (6 pairs), FRR for the fusion of signature and gaze (6 pairs). All other values are the same when a single curve is visualized.

**Figure 14:**
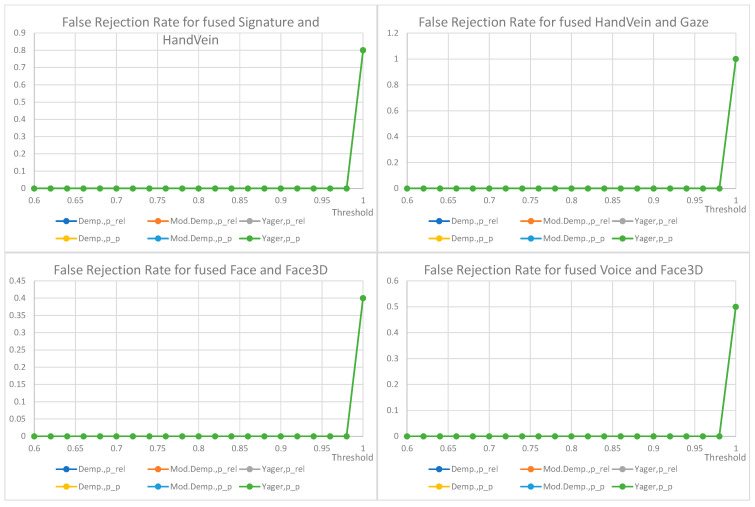
FRR for the fusion of signature and hand vein (5 pairs), FRR for the fusion of hand vein and gaze (5 pairs), FRR for the fusion of face and face3D (5 pairs), FRR for the fusion of voice and face3D (4 pairs). All other values are the same when a single curve is visualized.

**Figure 15:**
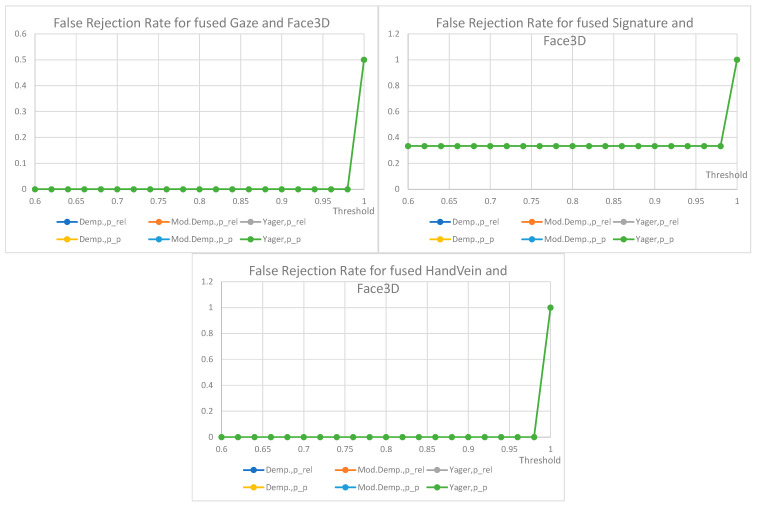
FRR for the fusion of gaze and face 3D (4 pairs), FRR for the fusion of signature and face 3D (3 pairs), FRR for the fusion of hand vein, and face3D (2 pairs). All other values are the same when a single curve is visualized.

False non-match rate (FNMR) for biometric identification verification based on the fusion of decisions from two traits is shown below (Figure 12, Figure 13, Figure 14 and Figure 15); the presentation order depends on the number of available and analyzed samples. On the plots, a jitter of 0.01 was added to the visualized datapoints values to distinguish between the data series better. It should be noted that all horizontal axes (threshold values) are presenting the scale [0.6–1.0], and the vertical axes ranges are automatically adjusted to cover the observed rejection rates values. On plots with a single green curve, all other results have the same value.

The plots show the cases when two given biometric modalities’ verification results are low enough to fall under the given decision threshold, and result in rejection. The higher the threshold is, the higher the requirement is for biometric comparison results. The lower the ratio, the better comparison results observed for the collected data. The used fusion methods are not distinguishable on the characteristics obtained for the number of pairs less than 13. However, in four cases where the number of biometric traits was larger than 13 (Figure 12), the general observation is that the Yager’s approach results in the highest false non-match rates, ca. 0.3 higher (Voice and Face fusion) or 0.2 higher (Signature and Voice fusion) than other approaches. The lowest FNMR occurs for Modified Dempster with pignistic probability among Dempster methods.

Among the three evaluated decision fusion methods, the Yager’s is generally the worst by means of the false non-match rates, although it is the most sensitive in cases of false identities. Therefore, this method is recommended to properly detect false identities for the weak and conflicting evidence, returning the lowest probabilities of false acceptance.

For the considered Yager’s method, the fused pair “voice and gaze” achieves the lowest rates of false rejections, “signature and face” being the second with FRR equal to 0.15 and 0.2, respectively, for a very high threshold of 0.98. Other fused modalities, namely “signature and voice” and “voice and face” achieve false rejection rates of 0.3 and 0.44 for the same threshold. For threshold above 0.92 the choice of mass to probability conversion method is irrelevant, but for lower values the relative probabilities result in higher FRRs than pignistic approach.

The choice of the final configuration: mass to probability conversion, fusion method, and decision threshold should be goal-oriented, and respective to the exploitation scenario. Several banking activities are typically made based on personal ID, some require a signature. Here, administrative decisions must define requirements on accepted target false rejections for given scenarios.

## 8. Conclusions

In the article, the design of a multimodal biometric verification system was presented, then three decision fusion methods, based on DST and comparison scores fusion, were presented and evaluated for synthetic and real biometric data from the system. It was shown that the fusion methods properly detect false identities for the weak and conflicting evidence. Yager’s approach dealt with conflicts in the most proper way, returning the lowest probabilities. However, false non-match rates are the highest for this method. The final setup of the system, the fusion method, and the decision threshold value should depend on the application requirements: the expected rates of false non-matches, false matches, and the scenario. Since the described biometric system was designed to operate under supervision in real banking branches, a rejected verification attempt can entail the intervention of a banking assistant.

Further work will aim to collect and process more multimodal samples in a real banking environment. For the pairs shown in Table 5, for which results were available, it was possible to demonstrate the usefulness of the developed methodology for performing biometric fusion. Hence, it seems reasonable to predict that these methods will also prove successful for other combinations of biometric modalities.

## Figures and Tables

**Table 1:** Summary of sensors for each stationary modality.

No.	Type of Modality	Sensor	Connection
1	Signature	Dynamic biometric pen	Bluetooth
2	Voice	Microphone	Internal MS Surface
3	Face image	External RGB camera integrated with 3D	USB-C
4	3D face image	External RGB camera integrated with 3D	USB-C
5	Gaze tracking	Gaze-tracker	USB 2.0
6	Hand vein pattern	Hand vein sensor	USB 2.0

**Table 2:** The probability estimation based on belief and plausibility values is based on known masses in the binary identity verification system.

Decision	Belief	Plausibility	Probability	Pignistic Probability	Relative Probability
Genuine	Bel(*g*) = m(*g*)	Pl(*g*) = m(*g*) + m(*e*)	Bel(*g*) ≤ p(*g*) ≤ Pl(*g*)	p_pign_(*g*) = m(*g*) + 0.5m(*e*)	p_rel_(*g*) = (1-m(*i*))/(1 + m(*e*))
Impostor	Bel(*i*) = m(*i*)	Pl(*i*) = m(*i*) + m(*e*)	Bel(*i*) ≤ p(*i*) ≤ Pl(*i*)	p_pign_(*i*) = m(*i*) + 0.5m(*e*)	p_rel_(*i*) = (1-m(*i*))/(1 + m(*e*))

**Table 3:** The number of samples collected for a given modality.

Modality	Number of Samples
Gaze	2047
Face	1034
Signature	790
Voice	759
Face3D	98
HandVein	16
Total:	4744

**Table 4:** Number of individuals registering at least the given number of different modalities from the set: Signature, Voice, HandVein, Face, Gaze, Face3D.

The Lowest Number *n* of Modalities Registered by the Single Individual in a Session	The Number of Individuals with at Least *n* Different Traits Registered
1	197
2	85
3	39
4	9
5	3
6	2

**Table 5:** Number of pairs of modalities acquired in a single session.

	Signature	Voice	HandVein	Face	Gaze	Face3D
Signature	-	48	5	56	6	3
Voice		-	6	79	13	4
HandVein			-	7	5	2
Face				-	13	5
Gaze					-	4
Face3D						-

## Data Availability

Not applicable.
